# Classification of Movement Intention Using Independent Components of Premovement EEG

**DOI:** 10.3389/fnhum.2019.00063

**Published:** 2019-02-22

**Authors:** Hyeonseok Kim, Natsue Yoshimura, Yasuharu Koike

**Affiliations:** ^1^Department of Information and Communications Engineering, Tokyo Institute of Technology, Yokohama, Japan; ^2^Institute of Innovative Research, Tokyo Institute of Technology, Yokohama, Japan; ^3^Precursory Research for Embryonic Science and Technology (PRESTO), Japan Science and Technology Agency (JST), Saitama, Japan

**Keywords:** brain-machine interface (BMI), electroencephalography (EEG), independent component analysis, classification, premovement

## Abstract

Many previous studies on brain-machine interfaces (BMIs) have focused on electroencephalography (EEG) signals elicited during motor-command execution to generate device commands. However, exploiting pre-execution brain activity related to movement intention could improve the practical applicability of BMIs. Therefore, in this study we investigated whether EEG signals occurring before movement execution could be used to classify movement intention. Six subjects performed reaching tasks that required them to move a cursor to one of four targets distributed horizontally and vertically from the center. Using independent components of EEG acquired during a premovement phase, two-class classifications were performed for left vs. right trials and top vs. bottom trials using a support vector machine. Instructions were presented visually (test) and aurally (condition). In the test condition, accuracy for a single window was about 75%, and it increased to 85% in classification using two windows. In the control condition, accuracy for a single window was about 73%, and it increased to 80% in classification using two windows. Classification results showed that a combination of two windows from different time intervals during the premovement phase improved classification performance in the both conditions compared to a single window classification. By categorizing the independent components according to spatial pattern, we found that information depending on the modality can improve classification performance. We confirmed that EEG signals occurring during movement preparation can be used to control a BMI.

## Introduction

Brain-machine interfaces (BMIs) are designed to decode neural commands from the brain and use them as input commands for external devices (Wolpaw et al., 2002; Höhne et al., 2014). Much of the development in BMIs has been to enable people with motor disabilities to interact with the external world (Mak and Wolpaw, 2009). Understanding brain activity associated with human intention leading to a movement task could further advance the effectiveness of BMIs as assistive devices.

Although there are many different methods for measuring brain activity, such as magnetoencephalography (MEG), electroencephalography (EEG), electrocorticography (ECoG), functional near-infrared spectroscopy (fNIRS; Naseer and Hong, 2015; Hong and Zafar, 2018), and functional magnetic resonance imaging (fMRI; Chaudhary et al., 2015), non-invasive methods offer practical advantages when used in BMIs. However, MEG and fMRI are generally unfit due to the scale of the equipment involved. Many researchers have tried to decode brain activity to understand human motor intention from EEG signals. Hybrid BMI such as EEG-fNIRS also has been tried for decoding (Hong and Khan, 2017; Khan and Hong, 2017; Hong et al., 2018). Linear decoding models have been used to predict upper limb kinematics during center-out reaching tasks (Úbeda et al., 2017), finger kinematics during reach to grasp movements (Agashe and Contreras-Vidal, 2011), and hand movement in three-dimensional space (Bradberry et al., 2010). However, the suitability of linear regression in modeling such tasks has been questioned (Antelis et al., 2013). Alternative modeling methods have also been applied. One study used a Kalman filter to estimate hand trajectory for a BMI (Robinson et al., 2015). Several studies on movement intention have also decoded brain activity as discrete information rather than continuous information, as in the studies cited above. Classification using discrete information has been performed for individual finger movements using a support vector machine (Liao et al., 2014), analytic movement tasks with the dominant upper limb (Ibáñez et al., 2015), as well as motor imagery for cursor control (Huang et al., 2009). Information on targets and movement direction may offer even greater versatility compared to that focusing on motor decoding. Several studies have classified movement direction (Hammon et al., 2008; Robinson et al., 2013) and targets (Shiman et al., 2017) during reaching tasks. Recently, combining EEG and fNIRS signals has been performed for early detection (Khan et al., 2018).

However, compared to the number of studies on brain activity during movement execution, only a few have attempted to classify the information before movement execution. Premovement brain activity has been used to detect movement intention during self-paced reaching tasks (Lew et al., 2012) as well as to predict targets (Novak et al., 2013), target direction (Hammon et al., 2008; Wang and Makeig, 2009), and hand kinematics (Yang et al., 2015). Moreover, studies on classification of movement direction typically used time windows that comprised target recognition after the target appeared (Hammon et al., 2008; Wang and Makeig, 2009). However, an interval before movement execution can be divided into two phases. The first phase comprises visual information. A person recognizes a target, which has information about movement direction. Then in the second phase, the person prepares to move the relevant body parts to execute movement. If the person does not wish to move immediately after target recognition, a gap may occur between target recognition and movement execution. If there is useful information for classification in this process, exploiting that information may provide improved capabilities in BMIs.

Therefore, in this study we investigated whether EEG signals before movement execution could be used to classify movement direction. We hypothesized that information from target recognition to movement can contribute to improvement of classification accuracy. Using independent components of EEG acquired during a premovement phase, two-class classifications were performed for left vs. right trials and top vs. bottom trials using a support vector machine. Instructions were presented visually (test) and aurally (control). In the test condition, accuracy for a single window was about 75%, and it increased to 85% in classification using two windows. In the control condition, accuracy for a single window was about 73%, and it increased to 80% in classification using two windows. Results showed that a combination of two windows from different time intervals during the premovement phase improved classification performance in the both conditions compared to a single window classification. We confirmed that EEG signals occurring during movement preparation can be used to control a BMI.

## Materials and Methods

### Experimental Procedure

Six healthy subjects (males, mean age ± standard deviation: 27.33 ± 1.51 years) participated in the experiment. This study was carried out in accordance with the recommendations of the ethics committees of Tokyo Institute of Technology (Ethics number: 2015062) with written informed consent from all subjects. All subjects gave written informed consent in accordance with the Declaration of Helsinki. The protocol was approved by the ethics committees of Tokyo Institute of Technology.

**Figure 1:**
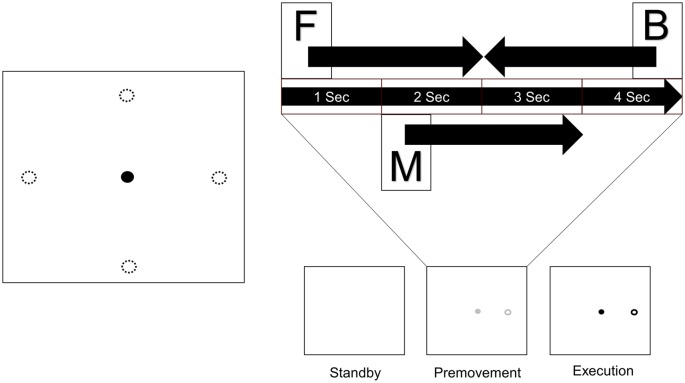
Experimental design. A target appeared at one of four positions distributed 4 cm from the center in the horizontal and vertical directions. Each trial consists of three phases. When a trial started, nothing appeared on the screen (Standby), and subjects waited for the next phase. Next, a cursor and target appeared on the screen, and subjects prepared for movement execution (Premovement). When the color of the markers changed to black, subjects moved the cursor from the center to the target using the touchpad (Execution). Three windows from the premovement phase were used for analysis (F: window starting at onset of the premovement phase, M: window starting 1 s after onset of the premovement phase, B: window before the execution phase). Four sizes were used for each window (0.5 s, 1.0 s, 1.5 s, and 2.0 s).

Subjects sat in a comfortable chair in front of a monitor on a desk with a touch pad. They adjusted their seat and the position of the touch pad to their comfort. After adjustment, they placed their hand on the touch pad and waited for onset of the experiment. Figure 1 shows the process of a trial. One trial consisted of three phases. In the first phase (standby phase), nothing appeared on the screen, and subjects waited for the next phase. In the second phase (premovement phase), a gray cursor and target appeared on the screen. The target appeared at one of four positions which were distributed 4 cm apart from the center in the horizontal and vertical directions. Subjects were instructed to recognize and prepare for movement execution, but not to perform it. This premovement phase lasted 4 s. In the final phase (execution phase), the color of the markers changed to black, cueing the subjects to move the cursor from the center to the target using the touchpad.

In the visual stimuli task, saccadic movements can dominantly influence classification regardless of the brain’s cortical process. This is unwanted information and should be removed. We provided auditory instruction as a control condition to remove this problem. As was done in the classifications with visual instruction, classifications were performed using a single window, two windows, and spatial categorization. In the control condition, the target was invisible. When the premovement phase began, auditory instructions (“left,” “right,” “up” and “down”) were provided. The eyes of the subjects were fixated to a cursor on the screen. When the execution phase began, a short beep sound was heard to signal the subjects to move to intended direction; and, no visual information was provided. All other procedures remained the same.

One run consisted of 40 trials, with 10 trials for each direction presented in random order. In the visual stimuli task, two of the subjects performed five runs, and the other four subjects performed three runs. In the auditory stimuli task, all of the subjects performed five runs. There was a rest after each run.

### Data Acquisition and Preprocessing

EEG data were acquired from 30 electrodes (FP1, FP2, AF3, AF4, F7, F8, F3, FZ, F4, FC5, FC6, T7, T8, C3, Cz, C4, CP5, CP6, P7, P8, P3, PZ, P4, PO7, PO8, PO3, PO4, O1, O2, A2) using a Quick-30 Dry EEG Headset (Cognionics, Inc., San Diego, CA, USA) designed according to the international 10–20 system (Klem et al., 1999). A2 was used as a reference electrode and the ground was placed at FPz. The data were sampled at 500 Hz.

EEGLAB (Delorme and Makeig, 2004) was used for preprocessing. EEG signals were high-pass filtered at 1.5 Hz and low-pass filtered at 4 Hz. Because the value at each time point was used as a feature, low frequency components were extracted. Cut-off frequency for high-pass filter was set for good ICA performance (Winkler et al., 2015). Epochs were extracted from the premovement phase. Noisy channels and trials were rejected by visual inspection. After that, independent component analysis was performed using the extended Infomax algorithm (Bell and Sejnowski, 1995) in EEGLAB. Extracted independent components showing noise were rejected.

**Figure 2:**
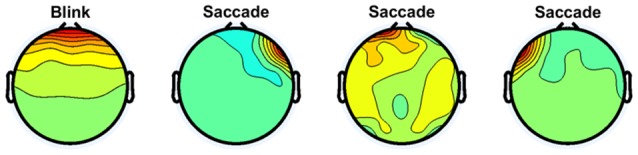
Independent components regarded as eye movement artifacts for subject 1.

Figure 2 shows examples of independent components regarded as eye movement artifacts for subject 1. The first independent component is a typical example of eye blinks. Similar components were observed in all subjects and rejected. The other independent components show examples of saccades. Because targets appeared leftward, rightward, upward, and downward from the center, saccades may have been included in the data. Each saccade involves activity at either the left or right frontal site (Plöchl et al., 2012). All independent components showing such patterns were rejected for all subjects.

### Classification Using a Single Temporal Window

Independent component analysis can divide EEG signals on the scalp into multiple electrical sources (Makeig et al., 1996). We applied independent component analysis after preprocessing to find sources that could be used as features for classification. However, because the data sampled at 500 Hz offered too many features and imposed excessive computational load, the data were down-sampled to 100 Hz using EEGLAB function. Two down-sampled independent components were used for classification. Classifications were performed separately for each window using all possible pairs of remaining independent components after rejecting components. If no components were rejected, classification was performed 10,440 times for one subject.

Since the purpose of this study was to examine the usability of EEG signals in the interval between target appearance and movement onset, only data in the premovement phase from three time windows were used (Figure 1). Window F comprised visual information right after target appearance. Window M was set such that it would not include visual information. Since target recognition varies between individuals, start time of window M was set to 1 s after target appearance. Window B was set such that it ended at onset of the execution phase. Each window took one of four window sizes (0.5 s, 1.0 s, 1.5 s, and 2.0 s).

Linear classifiers are widely used in BMI research (Lotte et al., 2007), and they are less vulnerable to overfitting (Müller et al., 2003). Of several linear classifiers, we employed support vector machine classifiers to perform binary classifications (left vs. right and top vs. bottom) because support vector machines offer strong generalization performance (Burges, 1998). Classification performance for each classifier was assessed using eight-fold cross-validation. Classification accuracy was calculated by subtracting the percentage of the sum of misclassifications for every test set from 100%. Using the Statistics and Machine Learning Toolbox in MATLAB version 9.2.0.556344 (R2017a, The MathWorks, Inc., Natick, MA, USA), support vector machine classifiers were implemented and classification performance was evaluated. Before computing classification performance, the random number generator was initialized to get the same result by applying the Mersenne Twister method (Matsumoto and Nishimura, 1998) with seed 1 (rng function in MATLAB).

### Secondary Classification Using Two Temporal Windows

Accuracies varied when using a single window for classification. Therefore, we further investigated whether combining information from different time windows would improve classification performance. Independent component pairs which provided classification accuracies greater than 65% for the single window classifier (chance level of 50%) were used in a second classifier. Classification was performed using a total of four independent components from two windows: one pair of components from window B and another pair from either window F or window M. For each selected pair, only window sizes which provided greater than 65% accuracy were used in the classifier. For example, if only the 0.5-s window F for a pair of first and second independent components gave a classification accuracy higher than 65%, pairs from the 1-s, 1.5-s, and 2-s windows F were not considered. As was done in classification using a single window, linear support vector machine classifiers were trained using all possible combinations of independent components from two windows. Evaluation of classification performance and all other procedures remained the same.

### Classification Following Spatial Categorization

**Figure 3:**
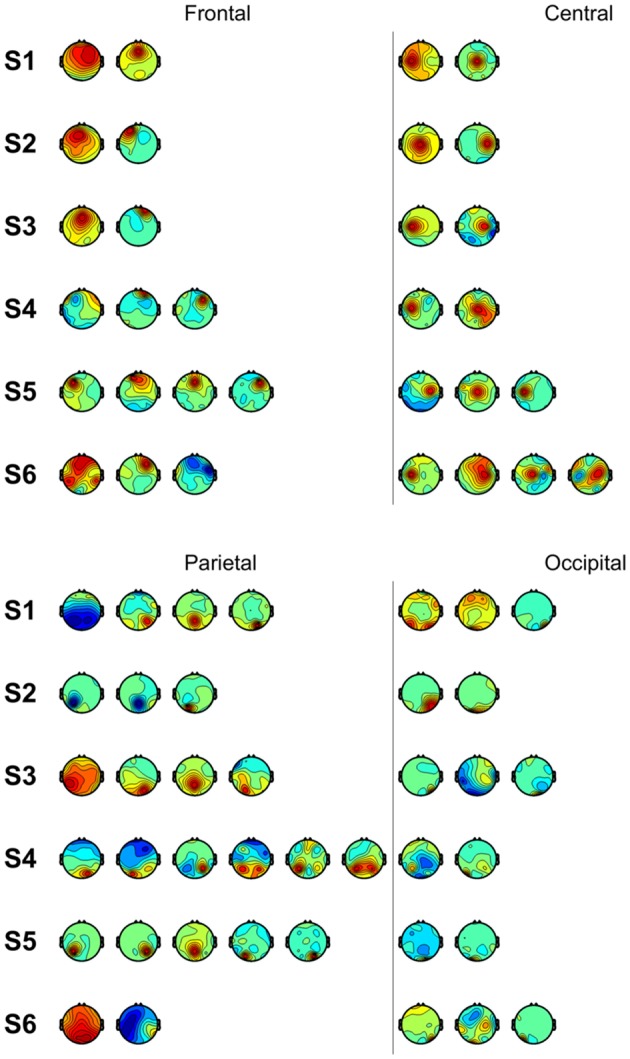
Scalp maps of independent components categorized according to area of peak activity for all subjects.

**Figure 4:**
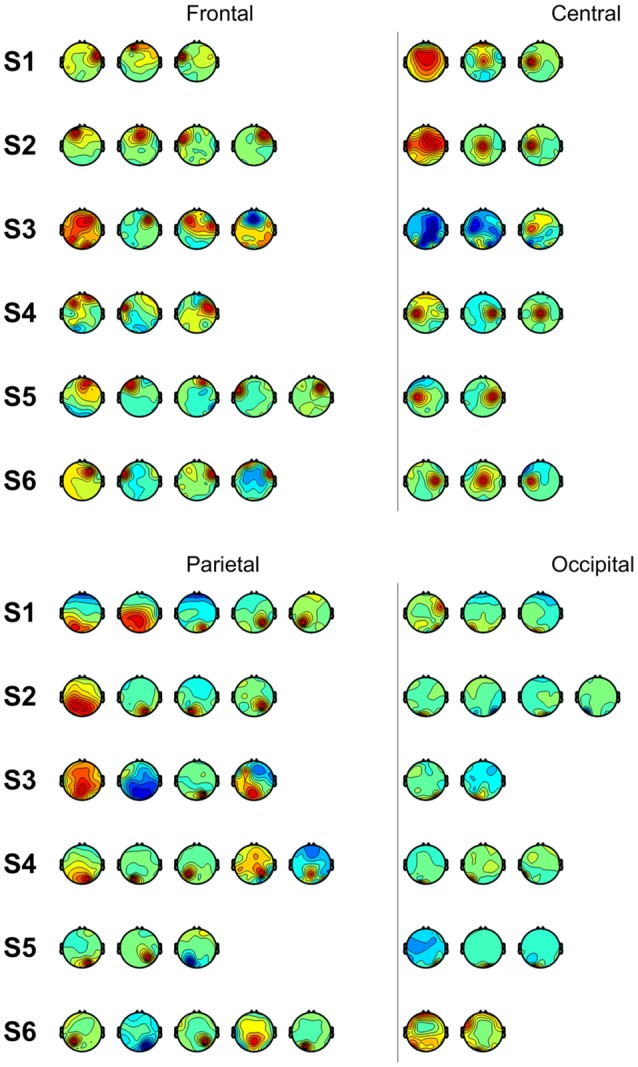
Scalp maps of independent components categorized according to area of peak activity for all subjects (control).

We also performed classification using independent components with similar spatial patterns for each subject. Figure 3 shows scalp maps of independent components categorized into four areas of peak activity for the test condition: frontal, central, parietal, and occipital. Figure 4 shows scalp maps for the control condition. The number of independent components with similar patterns was not the same for all subjects. Linear support vector machine classifiers were trained for all possible combinations of two independent components for each temporal window and brain area group. Two-class classifications (left vs. right and top vs. bottom) were performed, and classification performances were evaluated using the same procedure as that of the other classification methods.

## Results

In the case of left vs. right classification using a single window, all independent components that remained after noise rejection were used. Table 1 shows classification accuracies for left vs. right. Most classification accuracies were above 70% except for that of subject 3, window M (67.88%). The highest classification accuracy obtained was from subject 5, window B (87.25%). Mean classification accuracies were 76.46 ± 5.58%, 73.67 ± 3.94%, and 76.19 ± 5.77% for windows F, M, and B, respectively. Table 2 shows classification accuracies for left vs. right in the control condition. The lowest classification accuracy obtained was from subject 4, window B (67.09%). The highest classification accuracy obtained was from subject 6, window B (85.42%). Mean classification accuracies were 73.23 ± 5.24%, 73.53 ± 4.40%, 73.65 ± 6.60% for windows F, M, and B, respectively.

**Table 1:** Classification accuracies for left vs. right.

	Window position [%]				
	F	M	B	FB	MB
S1	73.72 (45.51)	73.72 (51.15)	73.72 (54.56)	85.90 (52.38)	85.26 (53.00)
S2	81.61 (49.46)	75.86 (49.07)	77.01 (48.87)	89.66 (48.01)	88.51 (54.59)
S3	70.91 (50.74)	67.88 (50.58)	75.15 (50.27)	85.45 (48.42)	81.21 (51.35)
S4	77.08 (48.97)	77.08 (50.27)	72.92 (49.87)	84.03 (49.78)	86.81 (50.74)
S5	84.31 (49.05)	77.45 (49.61)	87.25 (47.42)	95.10 (46.28)	97.06 (51.03)
S6	71.11 (48.80)	70.00 (49.40)	71.11 (50.41)	78.33 (47.26)	76.67 (51.47)
Mean	76.46 ± 5.58	73.67 ± 3.94	76.19 ± 5.77	86.41 ± 5.63	85.92 ± 6.93

*Values outside parenthesis are means of the three highest classification accuracies obtained among all independent component pairs. Values in parenthesis are means of accuracies when five inputs in shuffled conditions were fed to model which made values outside parenthesis. S indicates subject*.

**Table 2:** Classification accuracies for left vs. right (control).

	Window position [%]				
	F	M	B	FB	MB
S1	68.28 (50.74)	72.04 (49.73)	73.66 (51.48)	75.81 (49.37)	79.03 (50.33)
S2	71.43 (48.89)	70.37 (51.89)	75.66 (48.53)	83.07 (48.68)	81.48 (51.12)
S3	70.98 (50.47)	72.55 (50.83)	68.24 (49.17)	76.86 (51.93)	76.86 (46.67)
S4	71.79 (49.06)	70.94 (49.34)	67.09 (50.21)	73.93 (51.73)	70.94 (51.21)
S5	73.56 (48.84)	72.99 (52.57)	71.84 (50.13)	82.76 (49.14)	84.48 (48.99)
S6	83.33 (50.32)	82.29 (49.23)	85.42 (49.97)	90.63 (47.46)	92.71 (47.35)
Mean	73.23 ± 5.24	73.53 ± 4.40	73.65 ± 6.60	80.51 ± 6.21	80.92 ± 7.37

*Values outside parenthesis are means of the three highest classification accuracies obtained among all independent component pairs. Values in parenthesis are means of accuracies when five inputs in shuffled conditions were fed to model which made values outside parenthesis. S indicates subject*.

Left vs. right classification using two windows of independent component pairs (those with single window accuracies greater than 65%) resulted in higher accuracies than those using a single window (Table 1). Classification accuracies for subject 6 were 78.33% and 76.67%, using windows FB and MB, respectively. Classification accuracies for the other subjects were higher than 80%. Consistent with single window classification, the highest classification accuracy obtained was from subject 5 using window MB (97.06%). Mean classification accuracies were 86.41 ± 5.63% and 85.92 ± 6.93% for windows FB and MB, respectively. Accuracies for single window and double window showed significant difference (*p* < 0.01, paired *t*-test between window F and window FB; *p* < 0.01, paired *t*-test between window B and window FB; *p* < 0.01, paired *t*-test between window M and window MB; *p* < 0.01, paired *t*-test between window M and window MB). In the control condition, the highest classification accuracy obtained was from subject 6 using window MB (92.71%). Mean classification accuracies were 80.51 ± 6.21% and 80.92 ± 7.37% for windows FB and MB, respectively. Accuracies for single window and double window showed significant difference (*p* < 0.01, paired *t*-test between window F and window FB; *p* < 0.01, paired *t*-test between window B and window FB; *p* < 0.05, paired *t*-test between window M and window MB; *p* < 0.01, paired *t*-test between window M and window MB). We did not find significant differences between test and control in all windows (*p* > 0.1 for all cases). We shuffled conditions and extracted signals from it to apply input to models which achieved the highest classification accuracy. Values in parenthesis of Tables s 1, 2 show accuracies for models using random inputs in left vs. right classification. In all cases, accuracies were about 50%.

Table 3 shows classification accuracies for top vs. bottom. As with left vs. right classification, values shown for each subject are the mean of the three highest classification accuracies among those of all independent component pairs. All classification accuracies obtained were above 70%. The highest accuracy obtained was from subject 2, window F (86.21%). Mean classification accuracies were 76.78 ± 5.12%, 75.99 ± 4.12%, and 74.65 ± 4.17% for windows F, M, and B, respectively. Table 4 shows classification accuracies for top vs. bottom in the control condition. The lowest classification accuracy obtained was from subject 4, window F (67.98%). The highest classification accuracy obtained was from subject 6, window F and B (82.76%). Mean classification accuracies were 74.11 ± 5.46%, 74.06 ± 4.72%, 74.55 ± 5.00% for windows F, M, and B, respectively.

**Table 3:** Classification accuracies for top vs. bottom.

	Window position [%]				
	F	M	B	FB	MB
S1	70.99 (54.00)	72.84 (46.34)	74.07 (50.33)	80.86 (54.11)	88.27 (52.55)
S2	86.21 (52.03)	83.91 (48.89)	79.31 (46.24)	93.10 (49.73)	89.66 (49.12)
S3	75.33 (50.72)	74.00 (52.42)	71.33 (51.31)	83.33 (53.19)	86.00 (52.36)
S4	74.67 (52.03)	74.67 (50.02)	72.00 (50.24)	86.67 (50.63)	82.67 (52.14)
S5	77.78 (47.31)	76.92 (49.44)	80.34 (51.89)	93.16 (49.82)	90.60 (49.65)
S6	75.69 (54.41)	73.61 (46.44)	70.83 (52.62)	81.25 (54.42)	75.69 (49.55)
Mean	76.78 ± 5.12	75.99 ± 4.12	74.65 ± 4.17	86.40 ± 5.61	85.48 ± 5.58

*Values outside parenthesis are means of the three highest classification accuracies obtained among all independent component pairs. Values in parenthesis are means of accuracies when five inputs in shuffled conditions were fed to model which made values outside parenthesis. S indicates subject*.

**Table 4:** Classification accuracies for top vs. bottom (control).

	Window position [%]				
	F	M	B	FB	MB
S1	69.23 (48.98)	69.87 (45.88)	75.64 (49.33)	78.21 (49.07)	80.77 (49.94)
S2	72.58 (49.48)	71.51 (50.07)	69.89 (50.21)	77.42 (48.13)	77.42 (48.95)
S3	77.27 (44.44)	75.76 (47.70)	72.22 (48.43)	86.36 (49.56)	86.36 (47.88)
S4	67.98 (50.62)	69.30 (48.55)	69.74 (49.71)	70.18 (51.43)	74.12 (49.31)
S5	74.81 (50.34)	76.30 (48.11)	77.04 (50.01)	83.70 (48.41)	82.96 (48.83)
S6	82.76 (51.92)	81.61 (45.62)	82.76 (51.25)	91.95 (48.33)	93.10 (51.49)
Mean	74.11 ± 5.46	74.06 ± 4.72	74.55 ± 5.00	81.30 ± 7.66	82.46 ± 6.73

*Values outside parenthesis are means of the three highest classification accuracies obtained among all independent component pairs. Values in parenthesis are means of accuracies when five inputs in shuffled conditions were fed to model which made values outside parenthesis. S indicates subject*.

Top vs. bottom classification using two windows achieved higher accuracy than that using a single window (Table 3). The lowest classification accuracy obtained was from subject 6, window MB (75.69%). Classification accuracies for the other subjects were higher than 80%. Consistent with left vs. right classification, the highest classification accuracy obtained was from subject 5, window FB (93.16%). Mean classification accuracies were 86.40 ± 5.61% and 85.48 ± 5.58% for windows FB and MB, respectively. Accuracies for single window and double window showed significant difference (*p* < 0.01, paired *t*-test between window F and window FB; *p* < 0.01, paired *t*-test between window B and window FB; *p* < 0.01, paired *t*-test between window M and window MB; *p* < 0.01, paired *t*-test between window M and window MB). In the control condition, the highest classification accuracy obtained was from subject 6 using window MB (93.10%). The lowest classification accuracy obtained was from subject 4, window FB (70.18%). Mean classification accuracies were 81.30 ± 7.66% and 82.46 ± 6.73% for windows FB and MB, respectively. Accuracies for single window and double window showed significant difference (*p* < 0.01, paired *t*-test between window F and window FB; *p* < 0.05, paired *t*-test between window B and window FB; *p* < 0.01, paired *t*-test between window M and window MB; *p* < 0.01, paired *t*-test between window M and window MB). We did not find significant differences between test and control in all windows (*p* > 0.1 for all cases). Values in parenthesis of Tables 3, 4 show accuracies for models using random inputs in top vs. bottom classification. In all cases, accuracies were about 50%.

**Figure 5:**
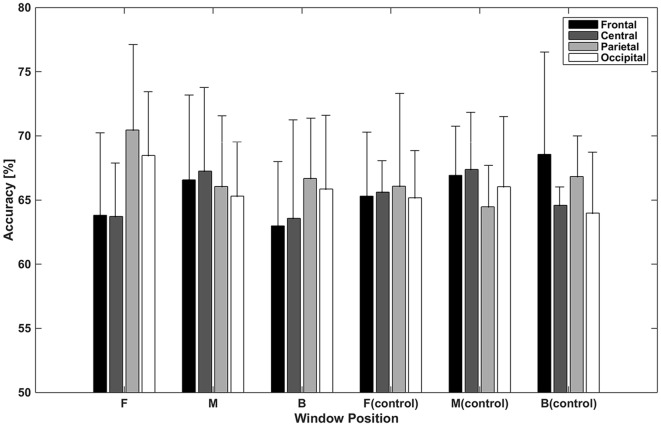
Classification accuracies using independent components categorized by spatial pattern. Values depicted are means of the highest accuracies obtained in left vs. right and top vs. bottom classifications averaged across subjects.

Figure 5 shows accuracies for classification using independent components categorized by spatial pattern. In the classifications using window F, accuracies for the parietal and occipital areas (70.46% and 68.47%, respectively) were higher than those for the frontal and central areas (63.82% and 63.72%, respectively). The difference between the former two and latter two accuracies was about 6% (*p* < 0.05, *t*-test between the former group and latter group), while accuracies within both were less than 1% (*p* > 0.05, *t*-test between the frontal and central area; *p* > 0.05, *t*-test between the parietal and occipital area). In the classifications using window M, the central area provided the highest accuracy (67.26%), while the frontal area provided the lowest (65.31%); however, the difference between them was small. In the classifications using window B, parietal area achieved the highest accuracy (66.68%). The frontal area showed the lowest performance (62.99%). In the case of the control condition, accuracies for the frontal area increased to 65.30%, 66.93%, and 68.57%. The central area showed a similar pattern in the test and control conditions. The classification using window M showed higher performance than that of window F or B. Unlike in the test condition, the difference between accuracies for the parietal and occipital areas and accuracies for the frontal and central areas was not high in window F.

## Discussion

In this study, we investigated utilization of premovement EEG signals to classify movement direction. Previous studies on classification of premovement EEG used brain activity during phases related to visual processes (Hammon et al., 2008; Wang and Makeig, 2009). Wang and Makeig (2009) reported a mean classification accuracy of 80.25%. In our results, accuracy using a single window was lower, at about 75%, but higher using two windows, at about 85%. Hammon et al. (2008) reported accuracies around 80% for left vs. right classification and 68% for top vs. bottom classification. However, an exact comparison cannot be made since their experimental tasks differed from ours, with targets appearing at one of four positions distributed diagonally from the center. They also used independent components as features for classification, but they set their window after the first 500 ms. We showed that combining windows in different time phase before movement execution and using independent components for each window could improve classification performance, which suggests that optimal components may exist for each time phase. Through our results, if we found the optimal spatial area suitable for each time phase, we could improve classification performance by selecting components strategically to understand human intention. Therefore, performances in previous studies might be improved by combining information in other time phases.

Under the control condition, which could exclude the possibility of saccadic movements, the two-window classifications showed higher accuracies than those of the single window classifications. Even when we used the windows which did not include recognition information, using both (the windows MB) showed higher accuracies compared to using either. Regardless of modalities, our result showed that combining independent components from different windows improves classification performance. In addition, we confirmed consistent performance regardless of modalities (*p* > 0.1 between test and control in all windows).

In the classifications using independent components categorized according to brain area, they showed high accuracies in the control condition, unlike the components from the frontal area which showed low accuracy in the test condition. As opposed to visual instructions which are intuitive, auditory instructions require subjects to remember and translate them. It has been reported that in verbal working memory, the prefrontal cortex showed greater activation during auditory stimuli in comparison to visual stimuli (Crottaz-Herbette et al., 2004). The central area showed a similar pattern in the test and control conditions. The accuracy for window F was higher than the other windows in both conditions. The central area contributes to accuracies regardless of the modality, but the central area did not show the highest accuracies for all windows. This implies that, though there are optimal independent components for classification according to the modality, if we sacrifice some accuracy, we can use less optimal components from the central area to achieve greater generalizability. Furthermore, because the central area includes the motor cortex, which is involved in motor planning (Li et al., 2015), this trend may reflect the motor area’s influence on motor preparation at each phase. The differences between accuracies for the visual stimuli and auditory stimuli were 4.38% (window F), 1.59% (window M) and −0.16% (window B) in the parietal area. The higher difference in window F may reflect that a wider parietal lobe is used to process visual stimuli compared to auditory stimuli (Poremba et al., 2003). In both conditions, the accuracies were small when the parietal area in window M was used. However, we cannot disregard the parietal area as unessential in window M. The parietal area is involved in motor planning (Cui and Andersen, 2007) and has been used for predicting movement intention (Wang and Makeig, 2009). Desmurget et al. (2009) reported that motor intention unconsciously leads to increased parietal activity. Components from the occipital area for windows F during the test task showed the highest accuracy in both conditions. Because target recognition takes up a large portion of the early phase, it is reasonable that the occipital area, which processes visual information, would provide high performance. This trend may reflect the availability of visual information at different phases. It is useful to exploit the occipital area using an early-phase window when visual stimuli are provided.

Although we removed components related to saccadic movements, the differences between accuracies in both conditions show that the accuracy is related to the modality. It implies that the brain’s activation related to stimuli can improve the accuracy. In goal directed tasks, movement is planned in the extrinsic coordinate system, but the muscle activation requires neural commands planned in the intrinsic coordinate system (Sarlegna and Sainburg, 2009). It has been reported that transformation into the intrinsic coordinate system is related to proprioception (Sober and Sabes, 2003). This physiological process could conceivably allow for decoding direction of movement even before the movement has started. However, further study is needed to determine whether the independent components used here reflect information about coordinate system transformations.

We used only EEG signals for decoding and confirmed that combining windows can improve performance. However, combining not only time phases but also other non-invasive methods can improve classification accuracy (Hong and Khan, 2017). Hybrid BMI has been studied for decoding (Hong et al., 2018) and should be studied in more detail. Therefore, features for different phases also should be investigated in hybrid BMI.

In this study, we confirmed that combining windows from different premovement phases offers improved performance over that using a single window. We used pairs of windows for classification. However, combining three windows, with each related to a different phase in movement preparation, may offer better performance. Because the purpose of this study was to investigate availability of classifiable premovement EEG, the time range was divided coarsely. Further studies that include calibration to determine optimal brain areas and time ranges could offer further progress toward a practical BMI.

## Conclusion

In conclusion, we investigated whether EEG signals occurring before movement execution could be used to classify movement intention. The result showed combining windows from different time phases can improve classification accuracy rather than using single window. In addition, we found consistent performance in different modalities. Furthermore, by categorizing the independent components according to spatial pattern, we found that information depending on the modality can improve classification performance. We confirmed that EEG signals occurring during movement preparation can be used to control a BMI.

## Author Contributions

HK designed and performed the experiment and drafted the manuscript. NY supported the experiment and analyzed the data. YK analyzed the data and contributed to the manuscript and supervised the research.

## Conflict of Interest Statement

The authors declare that the research was conducted in the absence of any commercial or financial relationships that could be construed as a potential conflict of interest.

## References

[B1] Agashe H. A. Contreras-Vidal J. L. (2011). “Reconstructing hand kinematics during reach to grasp movements from electroencephalographic signals,” in Proceedings of the Annual International Conference of the IEEE Engineering in Medicine and Biology Society (Bostan, MA: EMBC), 5444–5447. 10.1109/IEMBS.2011.6091389 22255569

[B3] Antelis J. M. Montesano L. Ramos-Murguialday A. Birbaumer N. Minguez J. (2013). On the usage of linear regression models to reconstruct limb kinematics from low frequency EEG signals. PLoS One 8:e61976. 10.1371/journal.pone.0061976 23613992 PMC3629197

[B4] Bell A. J. Sejnowski T. J. (1995). An information-maximization approach to blind separation and blind deconvolution. Neural Comput. 7, 1129–1159. 10.1162/neco.1995.7.6.1129 7584893

[B5] Bradberry T. J. Gentili R. J. Contreras-Vidal J. L. (2010). Reconstructing three-dimensional hand movements from noninvasive electroencephalographic signals. J. Neurosci. 30, 3432–3437. 10.1523/JNEUROSCI.6107-09.2010 20203202 PMC6634107

[B6] Burges C. J. C. (1998). A tutorial on support vector machines for pattern recognition. Data Min. Knowl. Discov. 2, 121–167. 10.1023/A:1009715923555

[B7] Chaudhary U. Birbaumer N. Curado M. (2015). Brain-machine interface (BMI) in paralysis. Ann. Phys. Rehabil. Med. 58, 9–13. 10.1016/j.rehab.2014.11.002 25623294

[B8] Crottaz-Herbette S. Anagnoson R. Menon V. (2004). Modality effects in verbal working memory: differential prefrontal and parietal responses to auditory and visual stimuli. Neuroimage 21, 340–351. 10.1016/j.neuroimage.2003.09.019 14741672

[B9] Cui H. Andersen R. A. (2007). Posterior parietal cortex encodes autonomously selected motor plans. Neuron 56, 552–559. 10.1016/j.neuron.2007.09.031 17988637 PMC2651089

[B10] Delorme A. Makeig S. (2004). EEGLAB: an open source toolbox for analysis of single-trial EEG dynamics including independent component analysis. J. Neurosci. Methods 134, 9–21. 10.1016/j.jneumeth.2003.10.009 15102499

[B11] Desmurget M. Reilly K. T. Richard N. Szathmari A. Mottolese C. Sirigu A. (2009). Movement intention after parietal cortex stimulation in humans. Science 324, 811–813. 10.1126/science.1169896 19423830

[B12] Hammon P. S. Makeig S. Poizner H. Todorov E. De Sa V. R. (2008). Predicting reaching targets from human EEG. IEEE Signal. Process. Mag. 25, 69–77. 10.1109/msp.2008.4408443

[B13] Höhne J. Holz E. Staiger-Sälzer P. Müller K.-R. Kübler A. Tangermann M. (2014). Motor imagery for severely motor-impaired patients: evidence for brain-computer interfacing as superior control solution. PLoS One 9:e104854. 10.1371/journal.pone.0104854 25162231 PMC4146550

[B14] Hong K.-S. Khan M. J. (2017). Hybrid brain-computer interface techniques for improved classification accuracy and increased number of commands: a review. Front. Neurorobot. 11:35. 10.3389/fnbot.2017.00035 28790910 PMC5522881

[B16] Hong K.-S. Khan M. J. Hong M. J. (2018). Feature extraction and classification methods for hybrid fNIRS-EEG brain-computer interfaces. Front. Hum. Neurosci. 12:246. 10.3389/fnhum.2018.00246 30002623 PMC6032997

[B15] Hong K.-S. Zafar A. (2018). Existence of initial dip for BCI: an illusion or reality. Front. Neurorobot. 12:69. 10.3389/fnbot.2018.00069 30416440 PMC6212489

[B17] Huang D. Lin P. Fei D.-Y. Chen X. Bai O. (2009). Decoding human motor activity from EEG single trials for a discrete two-dimensional cursor control. J. Neural Eng. 6:046005. 10.1088/1741-2560/6/4/046005 19556679

[B18] Ibáñez J. Serrano J. I. del Castillo M. D. Minguez J. Pons J. L. (2015). Predictive classification of self-paced upper-limb analytical movements with EEG. Med. Biol. Eng. Comput. 53, 1201–1210. 10.1007/s11517-015-1311-x 25980505

[B20] Khan M. J. Ghafoor U. Hong K-S. (2018). Early detection of hemodynamic responses using EEG: a hybrid EEG-fNIRS study. Front. Hum. Neurosci. 12:479. 10.3389/fnhum.2018.00479 30555313 PMC6281984

[B19] Khan M. J. Hong K.-S. (2017). Hybird EEG-fNIRS-based eight command decoding for BCI: application to quadcopter control. Front. Neurorobot. 11:6. 10.3389/fnbot.2017.00006 28261084 PMC5314821

[B21] Klem G. H. Lüders H. O. Jasper H. Elger C. (1999). The ten-twenty electrode system of the International Federation. Electroencephalogr. Clin. Neurophysiol. Suppl. 52, 3–6. 10590970

[B22] Lew E. Chavarriaga R. Silvoni S. Millán Jdel R. (2012). Detection of self-paced reaching movement intention from EEG signals. Front. Neuroeng. 5:13. 10.3389/fneng.2012.00013 23055968 PMC3458432

[B23] Li N. Chen T.-W. Guo Z. V. Gerfen C. R. Svoboda K. (2015). A motor cortex circuit for motor planning and movement. Nature 519, 51–56. 10.1038/nature14178 25731172

[B24] Liao K. Xiao R. Gonzalez J. Ding L. (2014). Decoding individual finger movements from one hand using human EEG signals. PLoS One 9:e85192. 10.1371/journal.pone.0085192 24416360 PMC3885680

[B25] Lotte F. Congedo M. Lécuyer A. Lamarche F. Arnaldi B. (2007). A review of classification algorithms for EEG-based brain-computer interfaces. J. Neural Eng. 4, R1–R13. 10.1088/1741-2560/4/2/R01 17409472

[B26] Mak J. N. Wolpaw J. R. (2009). Clinical applications of brain-computer interfaces: current state and future prospects. IEEE Rev. Biomed. Eng. 2, 187–199. 10.1109/rbme.2009.2035356 20442804 PMC2862632

[B27] Makeig S. Bell A. J. Jung T.-P. Sejnowski T. J. (1996). “Independent component analysis of electroencephalographic data,” in Advances in Neural Information Processing Systems, eds Touretzky D. Mozer M. Hasselmo M. (Cambridge, MA: MIT Press), 145–151.

[B28] Matsumoto M. Nishimura T. (1998). Mersenne twister: a 623-dimensionally equidistributed uniform pseudo-random number generator. ACM Trans. Model. Comput. Simul. 8, 3–30. 10.1145/272991.272995

[B29] Müller K.-R. Anderson C. W. Birch G. E. (2003). Linear and nonlinear methods for brain-computer interfaces. IEEE Trans. Neural Syst. Rehabil. Eng. 11, 165–169. 10.1109/tnsre.2003.814484 12899264

[B30] Naseer N. Hong K.-S. (2015). fNIRS-based brain-computer interfaces: a review. Front. Hum. Neurosci. 9:3. 10.3389/fnhum.2015.00003 25674060 PMC4309034

[B31] Novak D. Omlin X. Leins-Hess R. Riener R. (2013). Predicting targets of human reaching motions using different sensing technologies. IEEE Trans. Biomed. Eng. 60, 2645–2654. 10.1109/tbme.2013.2262455 23674417

[B32] Plöchl M. Ossandón J. P. König P. (2012). Combining EEG and eye tracking: identification, characterization, and correction of eye movement artifacts in electroencephalographic data. Front. Hum. Neurosci. 6:278. 10.3389/fnhum.2012.00278 23087632 PMC3466435

[B33] Poremba A. Saunders R. C. Crane A. M. Cook M. Sokoloff L. Mishkin M. (2003). Functional mapping of the primate auditory system. Science 299, 568–572. 10.1126/science.1078900 12543977

[B34] Robinson N. Guan C. Vinod A. (2015). Adaptive estimation of hand movement trajectory in an EEG based brain-computer interface system. J. Neural Eng. 12:066019. 10.1088/1741-2560/12/6/066019 26501230

[B35] Robinson N. Guan C. Vinod A. Ang K. K. Tee K. P. (2013). Multi-class EEG classification of voluntary hand movement directions. J. Neural Eng. 10:056018. 10.1088/1741-2560/10/5/056018 24018330

[B36] Sarlegna F. R. Sainburg R. L. (2009). “The roles of vision and proprioception in the planning of reaching movements,” in Progress in Motor Control. Advances in Experimental Medicine and Biology, ed. Sternad D. (Boston: Springer), 317–335. 10.1007/978-0-387-77064-2_16 PMC3709263 19227507

[B37] Shiman F. López-Larraz E. Sarasola-Sanz A. Irastorza-Landa N. Spüler M. Birbaumer N.. (2017). Classification of different reaching movements from the same limb using EEG. J. Neural Eng. 14:046018. 10.1088/1741-2552/aa70d2 28467325

[B38] Sober S. J. Sabes P. N. (2003). Multisensory integration during motor planning. J. Neurosci. 23, 6982–6992. 10.1523/JNEUROSCI.23-18-06982.2003 12904459 PMC6740676

[B39] Úbeda A. Azorín J. M. Chavarriaga R. Millán Jdel R. (2017). Classification of upper limb center-out reaching tasks by means of EEG-based continuous decoding techniques. J. Neuroeng. Rehabil. 14:9. 10.1186/s12984-017-0219-0 28143603 PMC5286813

[B40] Wang Y. Makeig S. (2009). “Predicting intended movement direction using EEG from human posterior parietal cortex,” in Foundations of Augmented Cognition. Neuroergonomics and Operational Neuroscience. FAC 2009. Lecture Notes in Computer Science, eds Schmorrow D. D. Estabrooke I. V. Grootjen M. (Berlin, Heidelberg: Springer), 437–446.

[B41] Winkler I. Debener S. Müller K. R. Tangermann M. (2015). “On the influence of high-pass filtering on ICA-based artifact reduction in EEG-ERP,” in Proceedings of the 37th Annual International Conference of the IEEEE ngineering in Medicine and Biology Society, (EMBC) (Milan: IEEE), 4101–4105. 10.1109/EMBC.2015.7319296 26737196

[B42] Wolpaw J. R. Birbaumer N. McFarland D. J. Pfurtscheller G. Vaughan T. M. (2002). Brain-computer interfaces for communication and control. Clin. Neurophysiol. 113, 767–791. 10.1016/S1388-2457(02)00057-3 12048038

[B43] Yang L. Leung H. Plank M. Snider J. Poizner H. (2015). EEG activity during movement planning encodes upcoming peak speed and acceleration and improves the accuracy in predicting hand kinematics. IEEE J. Biomed. Health Inform. 19, 22–28. 10.1109/jbhi.2014.2327635 24893371

